# Association of beta-2-microglobulin and cardiovascular events and mortality: A systematic review and meta-analysis

**DOI:** 10.1016/j.atherosclerosis.2021.01.018

**Published:** 2021-03

**Authors:** Fanchao Shi, Luanluan Sun, Stephen Kaptoge

**Affiliations:** MRC/BHF Cardiovascular Epidemiology Unit, Department of Public Health and Primary Care, University of Cambridge, Cambridge, United Kingdom

**Keywords:** Beta-2-microglobulin, Cardiovascular diseases, Mortality, Meta-analysis

## Abstract

**Background and aims:**

Beta-2-microglobulin (B2M) has been suggested as an emerging biomarker for cardiovascular diseases (CVD), including coronary heart disease (CHD) and stroke, and mortality.

**Methods:**

Three databases were searched from inception to January 2, 2020, supplemented by scanning reference lists of identified studies. We identified studies that reported associations of baseline serum or plasma B2M and CVD incidence, CVD mortality, or CHD and stroke separately, in either general populations or patients with renal disease. Relative risks (RR) were extracted and harmonized to a comparison of the highest *versus* lowest third of the distribution of B2M, and the results were aggregated.

**Results:**

Sixteen studies (5 in general populations, and 11 in renal disease populations) were included, involving 30,988 participants and 5391 CVD events. Based on random-effects meta-analysis, the pooled adjusted RRs comparing the highest *versus* lowest third of the distribution of B2M were 1.71 (95%CI: 1.37–2.13) for CVD, 2.29 (1.51–3.49) for CVD mortality, 1.64 (1.14–2.34) for CHD, and 1.51 (1.28–1.78) for stroke, with little to high heterogeneity between studies (0.0% ≤ I^2^ ≤ 80.0%). The positive associations between B2M and risks of CVD outcomes remained broadly significant across subgroup analyses. Moreover, the pooled adjusted RRs were 2.51 (1.94–3.26; I^2^ = 83.7%) for all-cause mortality and 2.64 (1.34–5.23; I^2^ = 83.1%) for infectious mortality.

**Conclusions:**

Available observational data show that there are moderate positive associations between B2M levels and CVD events and mortality, although few studies have been conducted in general populations.

## Highlights

• Available epidemiological evidence shows that circulating B2M levels are moderately associated with CVD events and mortality.
• Future large-scale general population-based prospective studies and genetic studies are needed to assess the causality.

## Graphical abstract

**Figure:**
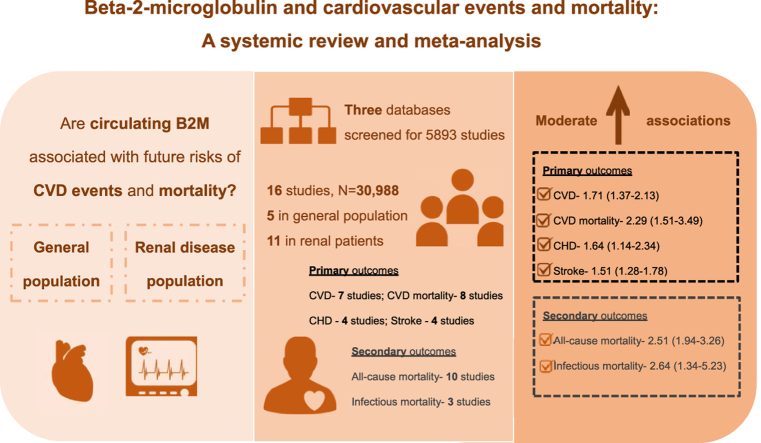

## 1 Introduction

Beta-2-microglobulin (B2M) first discovered in 1964 is a 100-amino acid protein (11.8 kDa) encoded by a gene in chromosome 15 in humans [1]. B2M is an important component of the major histocompatibility complex class I (MHC-I) molecule that is expressed on the surface of almost all nucleated cells [1]. B2M is necessary for the cell surface expression and structural stability of the MHC-I molecule [2], which plays key roles in antigen presentation and processing, inflammation, the complement cascade, and stress response [3,4]. B2M also complexes with many non-classical MHC-I like molecules such as CD1, MR1, HLA-E, –F, -G and neonatal Fc receptor [[5], [6], [7], [8]] that are involved in mucosal immunity, tumour surveillance, immunoglobulin and albumin homeostasis [9]. Moreover, B2M is constantly secreted into circulation from cell surfaces or intracellular release (0–3 mg/L concentration in plasma or serum) and is eliminated from blood predominantly by the kidneys under normal physiological conditions [2,10], and has particularly been studied as a biomarker of renal function [9].

In view of the high morbidity and mortality burden of cardiovascular diseases (CVD), there is great interest in discovering novel biomarkers that distinguish individuals at a higher risk of CVD [11]. Circulating B2M may be a potential biomarker given the associations of elevated B2M with inflammatory responses and declining glomerular filtration rate (GFR) [[12], [13], [14]], together with the involvement of inflammation and impaired GFR in the pathogenesis of vascular disease [[15], [16], [17], [18]]. Recent epidemiological studies have suggested higher B2M levels associated with higher CVD risk both in general populations studies [19], and in individuals with renal conditions [20]. However, the association between B2M and CVD has not yet been systematically assessed.

To address the above uncertainties, we conducted a systemic review and meta-analysis of published studies to primarily quantify the observational association of B2M and CVD outcomes, both in general populations and in renal patients; and investigated the associations of B2M with non-cardiovascular and all-cause mortality in the same cohorts.

## 2 Materials and methods

### 2.1 Search strategy and selection criteria

This review followed the guidelines in the MOOSE (Meta-analysis of Observational Studies in Epidemiology) statement [21]. A systematic search was conducted in PubMed, Web of Science and Embase databases from inception to January 2, 2020 for relevant studies reporting associations between B2M and CVD in general populations and people with renal diseases, motivated by the specific use of B2M as a renal biomarker [9]. The combined literature search terms were related to B2M and the outcomes (CVD or CVD mortality or CHD or cerebrovascular disease) with the restriction to English language (Supplementary Table 1). The literature search was complemented by reviewing reference lists of the identified studies.

Studies were eligible for inclusion if they met the following criteria: (1) full-length publication in English language available; (2) were prospective (nested case-control and prospective cohort studies) or retrospective (case-control and retrospective cohort studies) studies; (3) reported associations between baseline B2M (serum or plasma) and outcomes, i.e. CVD or CVD mortality or CHD (defined as non-fatal myocardial infarction, coronary heart disease death, or coronary revascularization) or stroke; (4) participants were primarily sampled from the general population or, secondarily, in renal disease populations. Studies that solely selected participants (in cohort studies) or controls (in nested case-control studies) on the basis of pre-existing CVD or metabolic abnormalities other than renal disease were excluded; (5) participants were adults (aged ≥18); (6) relative risk (RR) measures and corresponding 95% confidence interval (CI) were provided.

### 2.2 Data extraction and quality assessment

From each retrieved article, the following characteristics were extracted: name of first author, year of publication, study design, geographical location, data source, assay method, population type, proportion of female participants, age of participants, follow-up years, relevant outcome definitions, number of cases, mean and standard deviation of B2M, reported estimates of B2M association with outcome, scale of reported estimates, and degree of statistical adjustment for covariates. The estimate adjusted for conventional cardiovascular risk factors was chosen if more than one estimates were reported. Quality of the studies was assessed by Newcastle-Ottawa scale (NOS) [22] (Supplementary Data 2), by two reviewers (FS and LS) independently, and discussed with the third reviewer (SK). Study scores of 0–3, 4–6, and 7–9 were considered as low, moderate and high quality, respectively.

### 2.3 Statistics analysis

The overall associations between baseline B2M and CVD outcomes were estimated in cohorts or nested case-control studies. Hazard ratios, risk ratios, and odds ratios were assumed to approximate the same measure of RR on the basis of low incidence of the outcomes studied. When studies reported RRs only in subgroups (e.g. by sex), a single pooled estimate was first obtained for the study using fixed-effect meta-analysis. The study-specific relative risk estimates were transformed to correspond to a comparison of risk in the highest *versus* lowest third of the distribution of B2M using established methods [23] (further details provided in Supplementary Data 3). Non-cardiovascular and all-cause mortality in the selected studies were secondarily investigated.

A random-effects meta-analysis was conducted using the DerSimonian-Laird method to account for potential heterogeneity between studies. The heterogeneity between studies was assessed by Cochran's Q test and I^2^ statistics [24]. I^2^ statistics of <25%, 25–50%, 50–75% and >75% was considered as “no or little heterogeneity”, “low heterogeneity”, “moderate heterogeneity” and “high heterogeneity” respectively [24]. Due to the relatively small number of contributing studies, subgroup analyses were conducted based on random-effects meta-regression with hypothesis tests based on the t-distribution to explore study-level characteristics potentially explaining heterogeneity [25]. Funnel plots were used to assess publication or small study bias. Sensitivity analyses by omitting one study at a time were conducted to assess the influence of individual studies. To further evaluate whether renal function altered the results, analyses were conducted, where available, based on estimates adjusted for markers of renal function (e.g. estimated GFR (eGFR)), or restricted to the participants without chronic renal diseases (i.e. eGFR≥60 mL/min/1.73 m^2^). All analyses used Stata version 15.1 [26], and two-sided *p* < 0.05 was interpreted as statistically significant.

## 3 Results

### 3.1 Overall characteristics of selected studies

**Fig. 1:**
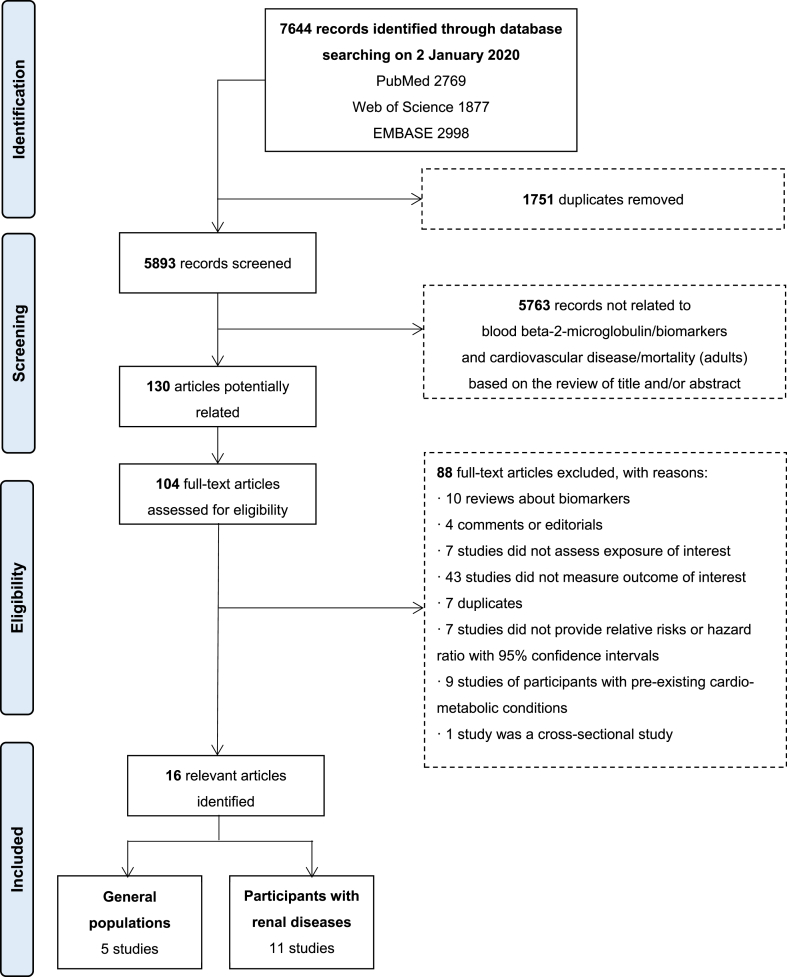
Literature review flow diagram. Matsushita et al. study [20], which primarily focused on CKD patients and was included into the 11 studies on participants with renal diseases here, also reported estimates for participants without CKD. CKD: chronic kidney disease.

Electronic searching from PubMed, Web of Science, and Embase identified 5893 relevant articles (Fig. 1). After a detailed assessment of 104 full-text available articles, 16 articles were included in this review. Table 1 summarises the characteristics of the studies included. In aggregate, 5391 cardiovascular events, including 1866 CVD mortality cases, 2352 CHD cases and 1257 stroke cases, were reported in fourteen prospective studies (28,486 participants), and two retrospective studies (2502 participants). Five studies were conducted in the general populations (in the United States (US)), while eleven studies were primarily conducted in participants with renal diseases (one in Europe; four in the US; and six in Asia), among which Matsushita et al. study [20] also reported estimates for people without chronic kidney disease (CKD). B2M was measured from plasma samples in four studies, and from serum samples in 12 studies. The overall quality assessed by NOS was relatively high (seven or more stars, with one study [27] having six stars) (Supplementary Table 2).

**Table 1:** Characteristics of 16 studies included in the review of the association between beta-2-microglobulin and cardiovascular disease.

Study	Study design	Region	Data source	Baseline survey	Population	B2M assay		Events for analysis	Sample size (female %)	Age (y)	Median follow-up (y)	B2M (mg/L) (mean ± SD)	No. of events			
						Ssource	Method						CVD	CVDM	CHD	Sstroke
General populations																
Astor, 2012 [39]	Prospective cohort	US	ARIC study	1990–1992	Community-based	Serum	PEINA (Siemens)	CHD	9988 (43.1)	62.9 ± 5.2^j^	10.2	2.1 ± 0.9^j^	–	–	1279	–
Foster, 2013 [33]	Prospective cohort	US	NHANES III Cystatin C project	1988–1994	Population-based	Serum	LA (Siemens)	CVDM	6445 (53.6)	≧20	14.4	1.8	–	1079	605^d^	–
								CHDM								
Prentice, 2013 [28]	Nested case-control	US	WHI HT trials	1993–1998	Postmenopausal women	Plasma	ELISA (CalBiotech)	CHD	710 (100.0)	50–79	7^c^	110^l^	–	–	358	362
								Stroke^h^	708 (100.0)							
Matsushita, 2014 [20]	Prospective cohort	US	ARIC study	1996–1998	Community-based (only non-CKDs)	Serum	PEINA (Siemens)	CVD	7682 (59)	62 ± 6	11.9	1.9 ± 0.4^j^	1336	–	–	277
								Stroke^i^								
Rist, 2017 [40]	Nested case-control	US	NHS	1989–1990	Female nurses	Plasma	ITA (Roche)	Ischaemic stroke	946 (100.0)	60.8 ± 6.0^j^	9.0	1.9 ± 0.4^m^	–	–	–	473
Ho, 2018 [19]	Prospective cohort	US	FHS	1998–2005	Community-based	Plasma	ELISA (Sigma-Aldrich)	CVD	3523 (53.3)	62 ± 8	14.3	NR	392^e^	167	–	–
								CVDM								
Renal disease populations																
Cheung, 2008 [34]	Prospective cohort	US	HEMO study	1995–2000	HD patients (ESRD)	Serum	RIA (Abbott)	CVDM	1813 (56.0)	57.6 ± 14.1	2.6^a^	37.6 ± 11.9	–	315^f^	–	–
Okuno, 2009 [35]	Prospective cohort	Japan	Hospital	1999	HD patients (ESRD)	Serum	LIA (Mitsubishi)	CVDM	490 (41.2)	60.1 ± 11.8	3.3^a^	32.5 ± 7.2	–	36	–	–
Liabeuf, 2012 [29]	Prospective cohort	France	Hospital	2006–2007	CKD stage 1–5 patients	Plasma	INA (Siemens)	CVD	142 (39.4)	67 ± 12	2.9	13.5 ± 12.5	49	24	–	–
								CVDM								
Astor, 2013 [36]	Retrospective cohort	US	Hospital	1996–2009	Kidney transplant recipients	Serum	MEIA (Abbott), ITA (Hitachi, Roche), NA (Siemens)	CVDM	2190 (40.3)	50.2 ± 13.0^j^	4.1	3.3	–	114	–	–
Matsushita, 2014 [20]	Prospective cohort	US	ARIC study	1996–1998	CKD stage 1–5 patients	Serum	PEINA (Siemens)	CVD	940 (59.5)	64.5 ± 5.5^j^	11.9	2.4 ± 0.7^j^	336	–	–	94
								Stroke^i^								
Matsui, 2016 [27]	Prospective cohort	Japan	Medical university	2010	PD patients (ESRD)	Serum	NR	CVD	40 (37.5)	62.8 ± 12.3^j^	1.5	20.8 ± 10.3^j^	13	–	–	–
Foster, 2016 [30]	Prospective cohort	US	CRIC study	2005–2008	CKD stage 1–3 patients	Serum	NA (Siemens)	CVD	2405 (47.9)	56.0 ± 11.6	6	4.2 ± 2.2	292	–	110^g^	51
								MI								
								Stroke^i^								
Wu, 2017 [31]	Retrospective cohort	China (Taiwan)	Hospital	2009–2015	CKD stage 3–5 patients	Serum	MEIA (Abbott)	CVD	312 (38.1)	70.9 ± 18.0^j^	3.3^a^	53.1 ± 23.2^j^	27	–	–	–
Yamashita, 2018 [37]	Prospective cohort	Japan	Hospital	2012	HD patients (ESRD)	Serum	NR	CVDM	307 (38.8)	68 ± 13	2^b^	26.9 ± 6.4	–	25	–	–
Chang, 2019 [38]	Prospective cohort	Korea	Hospital	2006–2011	PD patients (ESRD)	Serum	LIA	CVDM	725 (44.4)	59.3 ± 13.9	3.2	9.6 ± 8.3	–	106	–	–
Nishimura, 2019 [32]	Prospective cohort	Japan	Hospital	2005	HD patients (ESRD)	Serum	NR	CVD	244 (48.4)	64 ± 11	4.7^a^	41.4 ± 4.7^k^	78	–	–	–

ARIC Study: Atherosclerosis Risk in Communities Study; B2M: Beta-2-microglobulin; CHD: Coronary Heart Disease; CHDM: CHD Mortality; CKD: Chronic Kidney Disease; CRIC Study: Chronic Renal Insufficiency Cohort Study; CVD: Cardiovascular Disease; CVDM: CVD Mortality; ELISA: Enzyme-linked immunosorbent assay; ESRD: End Stage Renal Disease; FHS: Framingham Heart Study; HD: Hemodialysis; HEMO Study: Hemodialysis Study; INA: Immunonephelometric assay; ITA: Immunoturbidimetric assay; LA: Latex assay; LIA: Latex immunoassay; MEIA: Microparticle enzyme immunoassay; MI: Myocardial Infarction; NA: Nephelometric assay; NHANES III: The Third National Health and Nutrition Examination Survey; NHS: Nurses' Health Study; NR: Not Reported; PD: Peritoneal Dialysis; PEINA: Particle-enhanced immunonephelometric assay; RIA: Radioimmunoassay; US: United States; WHI HT Trials: Women's Health Initiative postmenopausal hormone therapy trials.

a Mean.

b Maximum.

c Minimum.

d No. of CHD mortality.

e No. of atherosclerotic CVD.

f No. of cardiac death.

g No. of MI.

h Both haemorrhagic and ischaemic stroke were included but cases number of subtypes were not reported.

i Whether haemorrhagic or ischaemic stroke was not specified.

j Mean ± SD calculated using https://home.ubalt.edu/ntsbarsh/business-stat/otherapplets/Pooled.htm and/or http://www.math.hkbu.edu.hk/~tongt/papers/median2mean.html.

k Calculated and the unit of this figure from the original study is ng/mL.

l Geometric mean reported in control groups was 0.11 mg/mL and equalled to 110 mg/L;^m^ mean ± SD in the control group.

### 3.2 Associations of B2M and cardiovascular outcomes

**Fig. 2:**
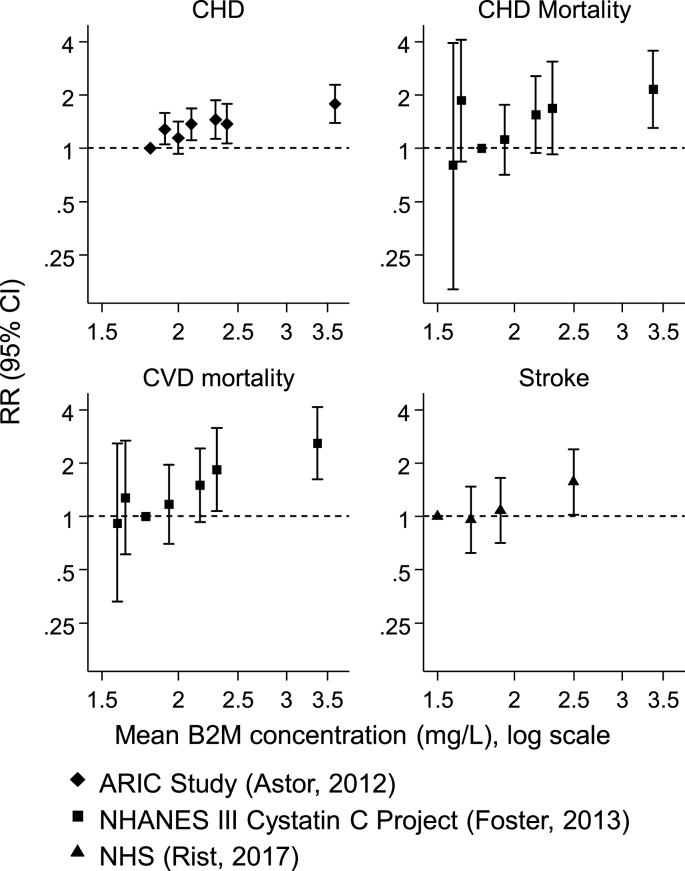
Relative risk of cardiovascular events according to categories of B2M levels for studies that provided results for quartiles or quintiles of B2M levels. ARIC Study: Atherosclerosis Risk in Communities Study; B2M: Beta-2-microglobulin; CVD: Cardiovascular Disease; CHD: Coronary Heart Disease; NHANES III: The Third National Health and Nutrition Examination Survey; NHS: Nurses' Health Study.

The RR estimates were converted into a comparison of highest *versus* lowest third of the distribution of B2M, except one study [28] where the standard deviation of B2M was not available (Supplementary Table 3). Fig. 2 shows the dose-response plots constructed for studies that used at least four categories of B2M levels, suggesting that a log-linear association of B2M and risk of CVD outcomes was reasonable.

#### 3.2.1 Cardiovascular disease outcomes

**Fig. 3:**
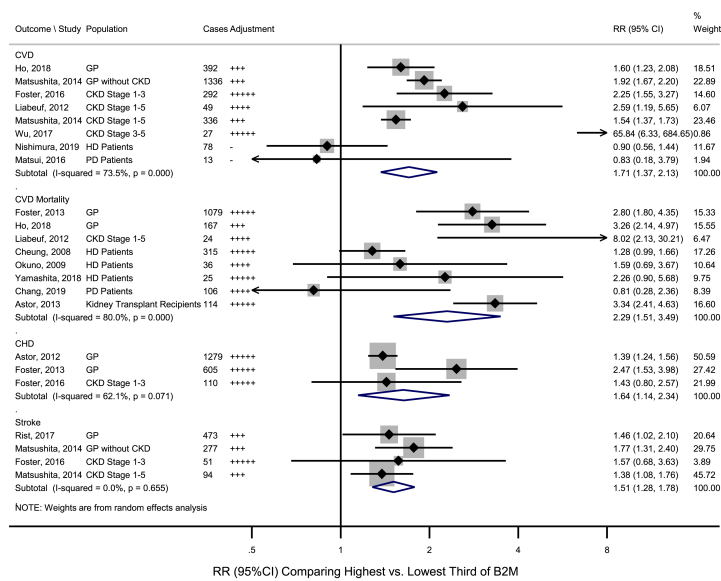
Association of B2M with risk for cardiovascular outcomes, comparing highest *versus* lowest third of B2M. HD/PD patients and those at CKD Stage 5 are normally ESRD patients. B2M: Beta-2-microglobulin; CHD: Coronary Heart Disease; CI: Confidence Interval; CKD: Chronic Kidney Disease; CVD: Cardiovascular Disease; ESRD: End Stage Renal Disease; GP: General Populations; HD: Hemodialysis; PD: Peritoneal Dialysis; RR: Relative Risk. Adjustment: no adjustment, + adjusted for age and/or sex, ++ age, sex, and non-lipid risk factors (e.g. race, medication use), +++ adjusted for age, sex, diabetes, body mass index/blood pressure/smoking and/or lipid markers, ++++adjusted for preceding plus inflammatory markers; +++++adjusted for preceding plus urinary indices.

Of the seven studies [19,20,27,[29], [30], [31], [32]] investigating the association between B2M and CVD, five [19,20,[29], [30], [31]] reported significant positive associations (Fig. 3 and Supplementary Table 3). The pooled RR for CVD comparing the highest *versus* lowest third of B2M was 1.71 (95%CI: 1.37–2.13; I^2^ = 73.5%, *p*_*het*_ < 0.001) (Fig. 3), and the pooled RR was 1.69 (1.33–2.14) for studies with further adjustments for renal function (Supplementary Figure 1). Sensitivity analysis omitting one study iteratively suggested that none of the included studies significantly influenced the pooled estimates, with RRs ranging from 1.63 (1.28–2.07) to 1.84 (1.49–2.26) (Supplementary Figure 2). The retrospective study by Wu et al. [31] reporting an RR of 65.84 (95%CI: 6.33–684.54) was considered as an outlier (meta-regression *p* = 0.034, by study design) (Table 2). Of the remaining prospective studies, the pooled RR was 1.66 (1.39, 1.99) with a moderate heterogeneity between studies (I^2^ = 64.7%, *p*_*het*_ = 0.009) (Table 2).

**Table 2:** Association of B2M with risk for CVD, CVD mortality, and all-cause mortality by recorded study level characteristics.

Subgroup	CVD					CVD mortality					All-cause mortality				
	No of studies^a^	No of cases	RR (95%CI)	I^2^ (%)	*p*_meta-regression_	No of studies	No of cases	RR (95%CI)	I^2^ (%)	*p*_meta-regression_	No of studies	No of cases	RR (95%CI)	I^2^ (%)	*p*_meta-regression_
All studies	8	2523	1.71 (1.37, 2.13)	73.5	–	8	1866	2.29 (1.51, 3.49)	80.0	–	10	6165	2.51 (1.94, 3.26)	83.7	–
Study design															
Retrospective	1	27	65.84 (6.33, 684.54)	–	0.034	1	114	3.34 (2.41, 4.63)	–	0.457	2	481	5.79 (1.06, 31.65)	58.5	0.315
Prospective	7	2496	1.66 (1.39, 1.99)	64.7		7	1752	2.13 (1.34, 3.37)	76.4		8	5684	2.36 (1.80, 3.09)	84.8	
Population															
General population	2	1728	1.82 (1.54, 2.14)	31.2	0.954	2	1246	3.03 (2.23, 4.11)	0	0.407	3	4572	2.00 (1.68, 2.39)	65.3	0.263
Renal disease patients	6	795	1.75 (1.13, 2.72)	76.2		6	620	2.04 (1.16, 3.59)	81.9		7	1593	2.94 (1.96, 4.39)	72.0	
Geographical location															
America	4	2356	1.75 (1.49, 2.05)	63.2	0.520	4	1675	2.47 (1.45, 4.20)	89.1	0.227	5	5677	2.56 (1.87, 3.49)	90.6	0.332
Asia	3	118	2.70 (0.37, 19.49)	84.0		3	167	1.51 (0.87.2.60)	3.3		4	444	2.04 (1.16, 3.61)	57.8	
Europe	1	49	2.59 (1.19, 5.66)	–		1	24	8.02 (2.13, 30.23)	–		1	44	6.61 (2.53, 17.28)	–	
Study quality (NOS)															
<8	3	140	1.30 (0.59, 2.83)	62.8	0.359	1	24	8.02 (2.13, 30.23)	–	0.155	1	44	6.61 (2.53, 17.28)	–	0.174
≧ 8	5	2383	1.83 (1.46, 2.28)	77.1		7	1842	2.11 (1.39, 3.21)	80.7		9	6121	2.39 (1.84, 3.09)	83.9	
Proportion of female participants															
<50%	5	459	2.06 (0.96, 4.42)	80.5	0.885	5	305	2.36 (1.30, 4.28)	62.7	0.899	8	3018	2.70 (1.80, 4.03)	87.0	0.660
≧50%	3	2064	1.69 (1.44, 1.98)	66.2		3	1561	2.23 (1.17, 4.23)	88.9		2	3147	2.18 (1.86, 2.56)	3.1	
Adjust for renal markers															
No	6	2204	1.59 (1.31, 1.92)	65.5	0.267	4	333	2.34 (1.09, 5.02)	69.4	0.939	4	1148	2.16 (1.36, 3.43)	66.1	0.498
Yes	2	319	9.91 (0.37, 264.28)	87.2		4	1533	2.26 (1.28, 3.98)	87.1		6	5017	2.78 (1.90, 4.08)	89.2	

B2M: Beta-2-microglobulin; CI: Confidence Interval; CVD: Cardiovascular disease; NOS: Newcastle-Ottowa Scale; RR: Relative Risk.

a Matsushita et al. study [20] was counted twice for CVD because estimates were provided for two populations, respectively.

#### 3.2.2 Cardiovascular mortality

Eight studies [19,29,[33], [34], [35], [36], [37], [38]] assessed the association between B2M and CVD mortality (Fig. 3 and Supplementary Table 3), with two [19,33] studies conducted in general populations reporting significant positive associations, and six [29,[34], [35], [36], [37], [38]] studies conducted in patients with renal diseases showing inconsistent associations. The pooled RR for CVD mortality comparing the highest *versus* lowest third of B2M was 2.29 (1.51–3.49; I^2^ = 80.0%, *p*_*het*_<0.001) (Fig. 3). Further adjustments for estimated renal function did not alter the results (Supplementary Figure 1). Subgroup and meta-regression analyses by study-level characteristics did not identify characteristics explaining heterogeneity (Table 2 and Supplementary Figure 4). Sensitivity analysis omitting one study at a time suggested that none of the individual studies significantly influenced the pooled estimates (Supplementary Figure 2).

#### 3.2.3 Coronary heart disease and stroke

Four studies reported associations with B2M on CHD [28,[30], [33],^39^] and stroke [20,28,30,40], respectively, which were all significantly positive in general populations. The pooled RR comparing the highest *vs* lowest thirds of B2M distribution was 1.64 (1.14–2.34; I^2^ = 62.1%, *p*_*het*_ = 0.071) for CHD, and 1.51 (1.28–1.78; I^2^ = 0.0%, *p*_*het*_ = 0.655) for stroke (Fig. 3), which remained significant with further adjustment for renal function or restricted to those with eGFR≥60 mL/min/1.73 m^2^ (Supplementary Figure 1 and 3). One study [28] was not included in the present meta-analysis due to inability to convert reported RRs (Supplementary Table 3), in which, 30% higher B2M was associated with RRs of 1.21 (1.06–1.37) for CHD, and 1.46 (1.21–1.78) for stroke, respectively.

### 3.3 Associations of B2M with non-cardiovascular and all-cause mortality

**Fig. 4:**
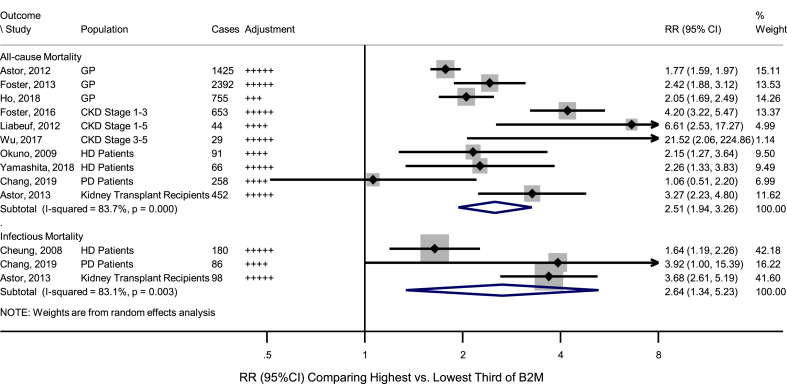
Association of B2M with risk for all-cause and infectious mortality, comparing highest *versus* lowest third of B2M. HD/PD patients and those at CKD Stage 5 are normally ESRD patients. B2M: Beta-2-microglobulin; CI: Confidence Interval; CKD: Chronic Kidney Disease; ESRD: End Stage Renal Disease; GP: General Populations; HD: Hemodialysis; PD: Peritoneal Dialysis; RR: Relative Risk. Adjustment: no adjustment, + adjusted for age and/or sex, ++ age, sex, and non-lipid risk factors (e.g. race, medication use), +++ adjusted for age, sex, diabetes, body mass index/blood pressure/smoking and/or lipid markers, ++++adjusted for preceding plus inflammatory markers; +++++adjusted for preceding plus urinary indices.

Among the included studies, 6165 all-cause mortality cases were reported by ten studies[19,[29], [30], [31],^33^,[35], [36], [37], [38], [39]], about 22.1%–54.5% of which were cardiovascular mortality; meanwhile, 364 infectious mortality cases were reported by three [34,36,38] studies (Supplementary Table 4). The pooled RR comparing the highest *vs* lowest thirds of B2M distribution was 2.51 (1.94–3.26; I^2^ = 83.7%, *p*_*het*_ <0.001) for all-cause mortality and 2.64 (1.34–5.23; I^2^ = 83.1%, *p*_*het*_ = 0.003) for infectious mortality (Fig. 4), which remained significant in all the sensitivity analyses (Supplementary Figure 1-3).

### 3.4 Publication bias

Since limited numbers of studies were available for each outcome, only CVD, CVD mortality and all-cause mortality studies (8, 8, and 10 estimates reported, respectively) were deemed suitable for the assessment of publication or small study bias using funnel plots. There was little evidence of publication or small study bias (Egger's test *p* > 0.05 and Begg's test *p* > 0.05 for all) among the studies of CVD, CVD mortality and all-cause mortality (Supplementary Figure 5).

## 4 Discussion

By integrating observational evidence from 16 studies, including 30,988 participants and 5391 CVD events, we primarily found positive associations of higher B2M levels and CVD outcomes, independent of conventional CVD risk factors as well as renal function; and secondarily found higher B2M levels were associated with increased risks of infectious and all-cause mortality. The associations between higher B2M levels and increased risk of CVD events and mortality persisted and remained broadly significant across study-level characteristics.

Previous individual studies on the associations of B2M and CVD outcomes have reported inconsistent results, with either positive [19,20,[28], [29], [30], [31],^33^,^36^,^39^,^40^] or no statistically significant [27,32,34,35,37,38] associations between B2M levels and CVD outcomes. In individual studies comprising models with different degrees of adjustment, the estimates of the association between B2M and cardiovascular outcomes were attenuated towards null [30,40] when further adjusting for eGFR. The present meta-analysis found significant associations between higher B2M levels and increased risks of CVD outcomes even after adjustment for inflammatory markers (e.g. albumin and C-reactive protein) and renal markers (e.g. eGFR), which were broadly consistent across the study-level characteristics assessed. The CVD and CVD mortality associations were somewhat stronger in general populations than in renal patients. In addition, the positive association of B2M and CVD, CHD or stroke appeared slightly stronger in individuals without chronic kidney disease than those with chronic kidney disease in one study [20]. The precise mechanisms linking B2M with CVD has not been fully understood, and it has been suggested that it may be partly due to renal function. B2M has been recognized as a marker of renal function [9], because it can be freely filtered by the glomerulus and reabsorbed and metabolized by the proximal tubule under normal kidney condition [2,10], and its circulating level rises when GFR declines [13]. Inflammation [12,13] has also been suggested as a potential mechanism linking B2M and CVD. Evidence suggested that higher B2M levels were positively associated with inflammatory markers [41].

Our meta-analysis found that B2M was also associated with all-cause mortality [19,[29], [30], [31],^33^,[35], [36], [37], [38], [39]] and mortality from infectious diseases [34,36,38] in the same cohorts, and the relevance of B2M with all-cause mortality was consistent across the study characteristics assessed in the present analyses. The positive associations with infectious and all-cause mortality were independent of renal function markers in line with previous findings in older age populations [42]. B2M has previously been found to be associated with other non-cardiovascular outcomes, such as various cancers [10], though not reported in many of the studies included in our review. Existing evidence has suggested that B2M is probably a general biomarker that reflects the acute or chronic changes during inflammation, infection, or immune dysregulation [2]. In our meta-analysis, however, compared to those for cardiovascular outcomes, the number of studies reporting B2M with non-cardiovascular outcomes was relatively limited, and the majority of study population patients had renal diseases [[29], [30], [31],[34], [35], [36], [37], [38]]. Hence, interpretations of the findings on B2M with non-cardiovascular mortality warrants cautions. This limitation emphasizes the need for prospective studies in general populations to compare the dose-response and magnitude of associations of B2M levels and incident disease outcomes.

B2M levels were markedly elevated with the progression of CKD and peaked in ESRD [29,36]. Compared to the general populations, the positive associations with CVD, CVD mortality and all-cause mortality seemed to be modest in ESRD patients undergoing dialysis [27,29,32,34,35,37,38]. These patients are special as high-flux dialysis could remove putatively atherogenic middle molecules [43,44] that may contribute to CVD events, such as advanced glycosylation end products [44]. Moreover, associations of B2M with CVD outcomes and all-cause mortality were found to be non-significant or even become significantly negative among patients that had undergone dialysis for over 3.7 years [34,45].

Although the majority of included studies comprehensively adjusted for potential confounders, the associations could still be subject to residual confounding or reserve causation bias, as our meta-analysis was based on observational studies. In the present meta-analysis, the association of B2M with CVD outcomes seemed to be attenuated according to some study level characteristics, such as smaller sample size and greater proportion of females, though no significant differences were found, which may be due to the low statistical power with few studies available. Longitudinal studies on changes of B2M demonstrated that B2M changes conveyed greater disease risk for CVD events [28,38]. While application of Mendelian randomization (MR) approaches may be informative (i.e. utilizing genotypes information fixed at conception to avoid reverse causation or confounding inherent in observational epidemiological studies [46]), we could not identify genetic instruments from currently available studies [47] that strictly fulfill assumptions underlying MR [48], thereby hampering further investigation. Hence, a comprehensive evaluation of associations in future well-powered genetic studies or randomized clinical trials of B2M and CVD are needed, given the evolving literature on B2M as potential drug targets [49,50] and gaps in translating research findings into clinical practice [15].

Our study has strengths. Our meta-analysis, by combining all available evidence so far, has provided improved statistical power than individual studies on the association between B2M levels and CVD outcomes. Our analyses were able to quantify the magnitude of the association between B2M and CVD outcomes, by harmonizing the reporting scales in individual studies, and to explore potential sources of heterogeneity between studies. Further, the linearity assumption underlying the RR conversions were satisfied based on checking studies that provided results by quartiles or quintiles of B2M levels.

Our study also had some limitations. First, the number of eligible studies identified reporting individual CVD outcomes was relatively small, in particular CHD, stroke, and stroke pathological types (e.g. ischaemic stroke and haemorrhagic stroke), reducing statistical power to detect heterogeneity [24]. Second, we only used aggregate data as reported or calculated in the published articles rather than analysis of individual participant data, thereby limiting the explorations of the contributions of individual level characteristics (e.g. observation time) to observed heterogeneity, or conducting a dose-response meta-analysis across all studies. Third, while publication or small study bias could in principle affect the results, it was not detected or possible to assess for all outcomes analysed in this study. Furthermore, the statistical tests concerning Begg's rank correlation and Egger's funnel plot asymmetry were less informative, given the small number of high-quality studies included [51]. Finally, our results only reflect the measurement of B2M at a single time point rather than longitudinal changes in B2M. Few studies [28,38,52] have so far explored the association between the change in B2M and cardiovascular diseases, although time-varying B2M demonstrated stronger associations with risk of CVD than baseline B2M [[28], [38]].

In summary, combined evidence from available observational studies shows positive associations between B2M level and risk of CVD outcomes, independent of conventional CVD risk factors, and estimated renal function. Future studies can help assess the causal nature of associations between B2M and CVD outcomes.

## Financial support

FS received funds from the China Scholarship Council, and Cambridge Commonwealth European and International Trust. LS and SK are supported by grant from the British Heart Foundation (BHF) (RG/18/13/33946).

## CRediT authorship contribution statement

**Fanchao Shi:** Study conception and design, literature search, inclusion, and statistical analysis, interpretation, first draft of manuscript, revision, All authors gave final approval and agree to be accountable for all aspects of work ensuring integrity and accuracy. **Luanluan Sun:** literature search, inclusion, and statistical analysis, interpretation, revision, All authors gave final approval and agree to be accountable for all aspects of work ensuring integrity and accuracy. **Stephen Kaptoge:** Study conception and design, literature search, inclusion, and statistical analysis, interpretation, revision, All authors gave final approval and agree to be accountable for all aspects of work ensuring integrity and accuracy.

## Declaration of competing interest

The authors declare that they have no known competing financial interests or personal relationships that could have appeared to influence the work reported in this paper.
