# Supplementary material for: Association of beta-2-microglobulin and cardiovascular events and mortality: A systematic review and meta-analysis
Source: Atherosclerosis. 2021 Mar;320:70–8. doi: 10.1016/j.atherosclerosis.2021.01.018 (PMC7955279; doi:10.1016/j.atherosclerosis.2021.01.018)
Supplement: Multimedia component 1 [file mmc1.pdf]

Appendix 1-4

They are intended for publication as an online data supplement.

Appendix 1. Search strategy

Supplementary Table 1. Literature search strategy for all the databases

PubMed, search through 2 January 2020	Results
(("beta 2-Microglobulin"[Mesh] OR "beta 2-Microglobulin" OR "beta-2 Microglobulin" OR "beta-2-Microglobulin" OR "beta 2 Microglobulin" OR "beta2-microglobulin" OR "B2M")) **AND** ("Cardiovascular Diseases"[Mesh] OR "Cardiovascular Diseases" OR "Cardiovascular Disease" OR "Cardiovascular Event" OR "Vascular Diseases" OR "Vascular Disease" OR "Ischemic Heart Disease" OR "Ischaemic Heart Disease" OR "Myocardial ischaemia" OR "Myocardial ischemia" OR "Acute coronary syndrome" OR "Coronary disease" OR "Coronary heart disease" OR "Coronary artery disease" OR "Coronary occlusion" OR "Coronary stenosis" OR "Coronary artery stenosis" OR "Cardiocerebrovascular disease" OR "Coronary thrombosis" OR "Myocardial infarction" OR "Heart attack" OR "Heart failure" OR "Cerebrovascular disease" OR "Cerebrovascular diseases" OR "Cerebrovascular disorder" OR "Cerebrovascular attack" OR "Stroke" OR "Apoplexy" OR "Brain vascular accident" OR "Cerebrovascular accident" OR "Cerebral Infarction" OR "Brain ischaemia" OR "Brain ischemia" OR "Ischaemic encephalopathy" OR "Ischemic encephalopathy" OR "Cardiovascular mortality" OR "Mortality"[Mesh] OR "all-cause mortality" OR "mortality" OR "Mortalities" OR "death"[Mesh] OR "death" OR "Risk"[Mesh] OR "Risk")	3128
Filter: English language	2769
**Web of Science, search through 2 January 2020**	**Results**
TS=("beta 2-Microglobulin" OR "beta-2 Microglobulin" OR "beta-2-Microglobulin" OR "beta 2 Microglobulin" OR "beta2-microglobulin" OR "β2-microglobulin" OR "B2M") **AND** TS=("Cardiovascular Diseases" OR "Cardiovascular Disease" OR "Cardiovascular Event" OR "Vascular Diseases" OR "Vascular Disease" OR "Ischemic Heart Disease" OR "Ischaemic Heart Disease" OR "Myocardial ischaemia" OR "Myocardial ischemia" OR "Acute coronary syndrome" OR "Coronary disease" OR "Coronary heart disease" OR "Coronary artery disease" OR "Coronary occlusion" OR "Coronary stenosis" OR "Coronary artery stenosis" OR "Cardiocerebrovascular disease" OR "Coronary thrombosis" OR "Myocardial infarction" OR "Heart attack" OR "Heart failure" OR "Cerebrovascular disease" OR "Cerebrovascular diseases" OR "Cerebrovascular disorder" OR "Cerebrovascular attack" OR "Stroke" OR "Apoplexy" OR "Brain vascular accident" OR "Cerebrovascular accident" OR "Cerebral Infarction" OR "Brain ischaemia" OR "Brain ischemia" OR "Ischaemic encephalopathy" OR "Ischemic encephalopathy" OR "Cardiovascular mortality" OR "all-cause mortality" OR "mortality" OR "Mortalities" OR "death" OR "Risk")	1938
Filter: English language	1877
**EMBASE, search through 2 January 2020**	**Results**
("beta 2-Microglobulin" or "beta-2 Microglobulin" or "beta-2-Microglobulin" or "beta 2 Microglobulin" or "beta2-microglobulin" or "β2-microglobulin" or "B2M").af. **AND** ("Cardiovascular Diseases" or "Cardiovascular Disease" or "Cardiovascular Event" or "Vascular Diseases" or "Vascular Disease" or "Ischemic Heart Disease" or "Ischaemic Heart Disease" or "Myocardial ischaemia" or "Myocardial ischemia" or "Acute coronary syndrome" or "Coronary disease" or "Coronary heart disease" or "Coronary artery disease" or "Coronary occlusion" or "Coronary stenosis" or "Coronary artery stenosis" or "Cardiocerebrovascular disease" or "Coronary thrombosis" or "Myocardial infarction" or "Heart attack" or "Heart failure" or "Cerebrovascular disease" or "Cerebrovascular diseases" or "Cerebrovascular disorder" or "Cerebrovascular attack" or "Stroke" or "Apoplexy" or "Brain vascular accident" or "Cerebrovascular accident" or "Cerebral Infarction" or "Brain ischaemia" or "Brain ischemia" or "Ischaemic encephalopathy" or "Ischemic encephalopathy" or "Cardiovascular mortality" or "all-cause mortality" or "mortality" or "Mortalities" or "death" or "Risk").af (Limited to Embase Status)	3136
Filter: English language	2998

Appendix 2. Newcastle-Ottowa Scale for quality assessment of studies[22]

Cohort studies: A study can be awarded a maximum of one star for each numbered item within the Selection and Outcome categories. A maximum of two stars can be given for Comparability.

Selection

1) Representativeness of the exposed cohort

   a) truly representative of general populations or renal disease populations in the community*

   b) somewhat representative of general populations or renal disease populations in the community*

   c) selected group of users e.g. nurses, volunteers

   d) no description of the derivation of the cohort

2) Selection of the non-exposed cohort

   a) drawn from the same community as the exposed cohort *

   b) drawn from a different source

   c) no description of the derivation of the non-exposed cohort

3) Ascertainment of exposure

   a) secure record (e.g. surgical records)*

   b) structured interview*

   c) written self-report

   d) no description

4) Demonstration that outcome of interest was not present at start of study

   a) yes*   b) no

Comparability

1) Comparability of cohorts on the basis of the design or analysis

   a) study controls for age*

   b) study controls for any additional factor*

Outcome

1) Assessment of outcome

   a) independent blind assessment*

   b) record linkage*

   c) self-report

   d) no description

2) Was follow-up long enough for outcomes to occur

   a) yes (median or mean follow-up≧6 years for general populations, ≧3 years for renal disease populations)*

   b) no

3) Adequacy of follow up of cohorts

   a) complete follow up - all subjects accounted for*

   b) subjects lost to follow up unlikely to introduce bias - small number lost - > 80 % follow up, or description provided of those lost*

   c) follow up rate < 80% and no description of those lost

   d) no statement

Appendix 3. Method used to transform relative risk estimates to common scale

The transformation assumed that the log relative risk was linear with B2M levels, or other reported transformation, and that the B2M or transformed B2M was normally distributed[23]. Taking the Foster et al study[33] as an example that provided estimates by quintiles, with quintile 3 as a reference and quintile 5 further split into 3 groups, a pooled estimate of 1.94 (95%CI: 1.46, 2.57) was first obtained from the combination of estimates for quintile 5a-5c. Given the standard normal distribution, the means of some quantile groups are shown below. The converted relative risk of 2.80 (95%CI: 1.80-4.35) was calculated based on the following reasoning: Assuming normality and log-linear association, the log relative risk for the highest versus lowest third of B2M is expected to correspond to 2.18 SD higher B2M, while the log relative risk for quintile 5 versus quintile 3 of B2M is expected to correspond to 1.40 SD higher B2M.

The multivariable-adjusted hazard ratios of CVD mortality by quintile of B2M compared to quintile 3

Quintile 1*	Quintile 2*	Quintile 4*	Quintile 5*		
			Quintile 5a	Quintile 5b	Quintile 5c
0.91 (0.33-2.57)	1.27 (0.61-2.69)	1.17 (0.70-1.96)	1.50 (0.93-2.41)	1.83 (1.07-3.14)	2.59 (1.62-4.14)

*Quintile 3 is the reference group with quintile 5 split into 3 equal groups. Extracted from Foster MC (2013, Table 3)[33].

-0.80	0.80					
-1.09	0	1.09				
-1.27	-0.32	0.32	1.27			
-1.40	-0.53	0	0.53	1.40		
-1.50	-0.68	-0.21	0.21	0.68	1.50	
-1.58	-0.80	-0.37	0	0.37	0.80	1.58

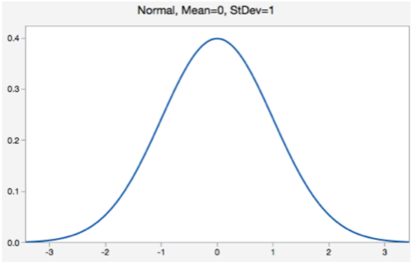

The means of some quantile groups, N(0,1). Under the assumption of normal distribution of B2M or transformed B2M and a log linear association with disease risk, the log relative risk for the highest versus lowest third of B2M is expected to correspond to 2.18 SD higher B2M; the log relative risk for quintile 5 versus quintile 3 of B2M is expected to correspond to 1.40 SD higher B2M.

The formulas to obtain the converted relative risk of 2.80 (95%CI: 1.80-4.35):

lnRR= ln(1.94)

SElnRR= (log upper relative risk- log lower relative risk)/(2*1.96)

Converted Relative Risk= exp(lnRR*2.18/1.40)

95%CI: (exp((lnRR-1.96*SElnRR)*2.18/1.40), exp((lnRR+1.96*SElnRR)*2.18/1.40))

Conversion factor= lncRR / lnRR

SElnRR: Standard error of log relative risk; lnRR: log relative risk; lncRR: log converted relative risk

Appendix 4. Results and discussion

Supplementary Table 2. Newcastle-Ottowa Scale quality assessment of included studies

	Study	Selection				Comparability	Outcome			Total (0-9)
		Representativeness of exposed cohort	Selection of non-exposed	Exposure ascertainment	Outcome not present at a start of study	Level of adjustment (analysis/ design)	Outcome assessment	Long enough follow-up	Adequate follow-up	
General Populations	Astor, 2012[39]	A (1*)	A (1*)	A (1*)	A (1*)	AB (2*)	B (1*)	A (1*)	B (1*)	9
	Foster, 2013[33]	A (1*)	A (1*)	A (1*)	A (1*)	AB (2*)	B (1*)	A (1*)	B (1*)	9
	Prentice, 2013[28]	C (0*)	A (1*)	A (1*)	A (1*)	AB (2*)	B (1*)	A (1*)	D(0*)	7
	Rist, 2017[40]	C (0*)	A (1*)	A (1*)	A (1*)	AB (2*)	B (1*)	A (1*)	D(0*)	7
	Ho, 2018[19]	A (1*)	A (1*)	A (1*)	A (1*)	AB (2*)	B (1*)	A (1*)	B (1*)	9
Renal Disease Populations	Cheung, 2008[34]	A (1*)	A (1*)	A (1*)	A (1*)	AB (2*)	B (1*)	B (0*)	B (1*)	8
	Okuno, 2009[35]	B (1*)	A (1*)	A (1*)	A (1*)	AB (2*)	B (1*)	A (1*)	A (1*)	9
	Liabeuf, 2012[29]	C (0*)	A (1*)	A (1*)	A (1*)	AB (2*)	B (1*)	B (0*)	A (1*)	7
	Astor, 2013[36]	B (1*)	A (1*)	A (1*)	B (0*)	AB (2*)	B (1*)	A (1*)	A (1*)	8
	Matsushita, 2014[20]	A (1*)	A (1*)	A (1*)	A (1*)	AB (2*)	B (1*)	A (1*)	B (1*)	9
	Matsui, 2016[27]	B (1*)	A (1*)	A (1*)	A (1*)	--- (0*)	B (1*)	B (0*)	A (1*)	6
	Foster, 2016[30]	A (1*)	A (1*)	A (1*)	A (1*)	AB (2*)	B (1*)	A (1*)	B (1*)	9
	Wu, 2017[31]	B (1*)	A (1*)	A (1*)	B (0*)	AB (2*)	B (1*)	A (1*)	B (1*)	8
	Yamashita, 2018[37]	B (1*)	A (1*)	A (1*)	A (1*)	AB (2*)	B (1*)	B (0*)	A (1*)	8
	Chang, 2019[38]	B (1*)	A (1*)	A (1*)	A (1*)	AB (2*)	B (1*)	A (1*)	B(1*)	9
	Nishimura, 2019[32]	B (1*)	A (1*)	A (1*)	A (1*)	--- (0*)	B (1*)	A (1*)	A (1*)	7

One star (1*) means 1 score. A study can be awarded a maximum of one star for each numbered item within the Selection and Outcome categories. A maximum of two stars can be given for Comparability. Study scores of 0-3, 4-6, and 7-9 were considered as low, moderate and high quality, respectively.

Supplementary Table 3. Further characteristics of cardiovascular outcomes of 16 studies included in the review

Study \ *Sub-analyses*	Population	RR (95%CI) reported	Scale of RR reported by study	*p* for trend if reported	Conversion factor [a]	RR (95%CI) in highest vs. lowest third of B2M	Adjustment[e]	Adjusted renal marker [g]
**CVD Studies**								
Ho, 2018[19]	GP	1.24 (1.10, 1.40)	per 1 SD increase in rank normalized data	NA	2.18	1.60 (1.23, 2.08)	+++	-
Matsushita, 2014[20]	GP (only non-CKDs)	1.35 (1.27, 1.44)	per 1 SD increase in log B2M	<0.001[h]	2.18	1.92 (1.68, 2.21)	+++	-
Liabeuf, 2012[29]	CKD stage 1-5	2.04 (1.13, 3.64)[b]	B2M >8.34 versus ≤8.34 mg/L	NA	1.34	2.59 (1.19, 5.66)	++++	-
Matsushita, 2014[20]	CKD stage 1-5	1.22 (1.16, 1.29)	per 1 SD increase in log B2M	<0.001[h]	2.18	1.54 (1.37, 1.73)	+++	-
Matsui, 2016[27]	PD patients	0.87 (0.28, 2.62)	B2M ≥18.1 versus <18.1 mg/L	NA	1.35	0.83 (0.18, 3.76)	-	-
Foster, 2016[30]	CKD stage 1-3	1.45 (1.22, 1.72)	1/B2M, per unit decrease in z score (1 SD in B2M)	NA	2.18	2.25 (1.55, 3.27)	+++++	UACR
Estimates with maximum adjustment [i]:		*1.69 (1.14, 2.51)*	*1/B2M, per unit decrease in z score (1 SD in B2M)*	*NA*	*2.18*	*3.14 (1.33, 7.43)*	*+++++*	*UACR+ mGFR*
Wu, 2017[31]	CKD stage 3-5	65.84 (6.33, 684.26)	highest tertile versus lowest tertile	NR	1.00	65.84 (6.33, 684.54)	+++++	eGFRcr
Nishimura, 2019[32]	HD patients	0.99 (0.94, 1.03)	per 1 ng/ml increase (2.5 SD)	NA	10.25	0.90 (0.56, 1.44)	-	-
**CVDM Studies**								
Foster, 2013[33]	GP	1.94 (1.46, 2.57)[c]	Quintile 5 versus Quintile 3	<0.001	1.56	2.80 (1.80, 4.35)	+++++	UACR
Subgroup: eGFRcr ≥60mL/min/1.73m^2		*1.70 (1.28, 2.25)*[c]	*Quintile 5 versus Quintile 3*	*0.001*	*1.56*	*2.28 (1.47, 3.55)*	*+++++*	*UACR*
Ho, 2018[19]	GP	1.72 (1.42, 2.09)	per 1 SD increase in rank normalized data	NA	2.18	3.26 (2.14, 4.98)	+++	-
Cheung, 2008[34]	HD patients	1.10 (0.99, 1.21)	per 10 mg/L increase (0.84 SD)	NA	2.60	1.28 (0.99, 1.66)	+++++	KRU
Okuno, 2009[35]	HD patients	1.03 (0.98, 1.09)	per 1 mg/L increase (0.14 SD)	NA	15.71	1.59 (0.69, 3.67)	++++	-
Liabeuf, 2012[29]	CKD stage 1-5	4.75 (1.76, 12.83)	B2M >8.34 versus ≤8.34 mg/L	NA	1.34	8.02 (2.13, 30.23)	++++	-
Astor, 2013[36]	KTR	4.70 (3.10, 7.19)[d]	highest versus lowest fifth	NR	0.78	3.34 (2.41, 4.64)	+++++[f]	eGFRcr
Yamashita, 2018[37]	HD patients	1.06 (0.99, 1.13)	per 1 mg/L increase (0.16 SD)	NA	13.96	2.26 (0.90, 5.68)	+++++	Urine volume
Chang, 2019[38]	PD patients	0.90 (0.53, 1.54)	highest tertile versus middle tertile	NR	2.00	0.81 (0.28, 2.35)	++++	-
Estimates with maximum adjustment [i]:		*0.72 (0.40, 1.30)*	*highest tertile versus middle tertile*	*NR*	*2.00*	*0.52 (0.16, 1.68)*	*+++++*	*eGFRcr*
**CHD Studies**								
Astor, 2012[39]	GP	1.53 (1.32, 1.77)[c]	highest versus lowest fifth	<0.001	0.78	1.39 (1.24, 1.56)	+++++	UACR
Subgroup: eGFRcr ≥60mL/min/1.73m^2		*1.35 (1.14, 1.59)*[c]	*highest versus lowest fifth*	*0.01*	*0.78*	*1.26 (1.11, 1.44)*	*+++++*	*UACR*
Foster, 2013[33]	GP	1.79 (1.31, 2.42)[c]	Quintile 5 versus Quintile 3	0.006	1.56	2.47 (1.53, 3.97)	+++++	UACR
Subgroup: eGFRcr ≥60mL/min/1.73m^2		*1.48 (1.07, 2.04)*[c]	*Quintile 5 versus Quintile 3*	*0.4*	*1.56*	*1.84 (1.11, 3.04)*	*+++++*	*UACR*
Prentice, 2013[28]	GP	1.21 (1.06, 1.37)	30% increase of baseline B2M	NA	NA	NA	++++	-
Foster, 2016[30]	CKD stage 1-3	1.18 (0.90,1.54)	1/B2M, per unit decrease in z score (1 SD in B2M)	NA	2.18	1.43 (0.80, 2.58)	+++++	UACR
Estimates with maximum adjustment [i]:		*1.89 (1.00,3.58)*	*1/B2M, per unit decrease in z score (1 SD in B2M)*	*NA*	*2.18*	*4.01 (1.00, 16.12)*	*+++++*	*UACR+ mGFR*

Study \ *Sub-analyses*	Population	RR (95%CI) reported	Scale of RR reported by study	*p* for trend if reported	Conversion factor [a]	RR (95%CI) in highest vs. lowest third of B2M	Adjustment[e]	Adjusted renal marker [g]
**Stroke Studies**								
Prentice, 2013[28]	GP	1.46 (1.21, 1.78)	30% increase of baseline B2M	NA	NA	NA	++++	-
Rist, 2017[40]	GP	1.56 (1.02, 2.39)	highest versus lowest forth	0.02	0.86	1.46 (1.02, 2.11)	+++	-
Estimates with maximum adjustment [i]:		*1.53 (0.98, 2.41)*	*highest versus lowest forth*	*0.04*	*0.86*	*1.44 (0.98, 2.12)*	*+++++[f]*	*eGFRcr*
Subgroup: eGFRcr ⩾60mL/min/1.73m²		*1.49 (1.08, 2.06)*	*highest versus lowest forth*	*NR*	*0.86*	*1.41 (1.07, 1.86)*	*+++*	*-*
Matsushita, 2014[20]	GP (only non-CKDs)	1.30 (1.13, 1.49)	per 1 SD increase in log B2M	NA	2.18	1.77 (1.31, 2.40)	+++	-
Matsushita, 2014[20]	CKD stage 1-5	1.16 (1.04, 1.30)	1per 1 SD increase in log B2M	NA	2.18	1.38 (1.08, 1.76)	+++	-
Foster, 2016[30]	CKD stage 1-3	1.23 (0.84,1.81)	1/B2M, per unit decrease in z score (1 SD in B2M)	NA	2.18	1.57 (0.68, 3.63)	+++++	UACR
Estimates with maximum adjustment [i]:		*1.27 (0.54,2.98)*	*1/B2M, per unit decrease in z score (1 SD in B2M)*	*NA*	*2.18*	*1.68 (0.26, 10.86)*	*+++++*	*UACR+ mGFR*

Note: HD/PD patients and those at CKD Stage 5 are normally ESRD patients.

B2M: Beta-2-microglobulin; CHD: Coronary Heart Disease; CI: Confidence Interval; CKD: Chronic Kidney Disease; CVD: Cardiovascular Disease; CVDM: CVD Mortality; eGFRcr: estimated Glomerular Filtration Rate based on creatinine; ESRD: End Stage Renal Disease; HD: Hemodialysis; KRU: residual kidney urea clearance; KTR: Kidney Transplant Recipients; mGFR: measured Glomerular Filtration Rate; NA: Not Applicable; NR: Not Reported; PD: Peritoneal Dialysis; RR: Relative Risk; UACR: Urine Albumin-to-Creatinine Ratio.

[a] To convert reported log RR to log RR in highest versus lowest third of the B2M distribution; [b] Converted based on the HRs of lnB2M (continuous variable); [c] Pooled estimated from Quintile 5a, 5b and 5c; [d] Converted based on the HR of a doubling of B2M; [e] -no adjustment, + adjusted for age and/or sex, ++ age, sex, and non-lipid risk factors (e.g. race, medication use) , +++ adjusted for age, sex, diabetes, body mass index/ blood pressure/ smoking and/or lipid markers, ++++adjusted for preceding plus inflammatory markers; +++++adjusted for preceding plus urinary indices.

[f] Adjustments do not include inflammatory markers.

[g] Renal markers (e.g. eGFRcr or UACR) in the adjustments.

[h] The p value for trend was also reported for RR in different quantiles though RR here was for continous variable.

[i] RR adjusted for conventional cardiovascular risk factors was chosen in priority if more than one estimates were reported, followed by RR with maximum adjustment.

Supplementary Table 4. Characteristics of infectious/non-cardiovascular and all-cause mortality of 16 studies included in the review

Study \ *Sub-analyses*	Population	Events/ N	RR (95%CI) reported	Scale of RR reported by study	*p* for trend if reported	Conversion factor [a]	RR (95%CI) in highest vs. lowest	Adjustment [e]
**All-cause Mortality Studies**								
Astor, 2012[39]	GP	1425/ 9988	2.08 (1.81, 2.38)[c]	highest versus lowest fifth	<0.001	0.78	1.77 (1.59, 1.97)	+++++
Subgroup: eGFRcr ≥60mL/min/1.73m²		*1201/9320*	*1.89 (1.63, 2.20)*[c]	*highest versus lowest fifth*	*<0.001*	*0.78*	*1.64 (1.46, 1.85)*	*+++++*
Foster, 2013[33]	GP	2392/ 6445	1.76 (1.50, 2.08)[c]	Quintile 5 versus Quintile 3	<0.001	1.56	2.42 (1.88, 3.12)	+++++
Subgroup: eGFRcr ≥60mL/min/1.73m²		*1734/ 5632*	*1.63 (1.37, 1.95)*[c]	*Quintile 5 versus Quintile 3*	*<0.001*	*1.56*	*2.15 (1.63, 2.82)*	*+++++*
Ho, 2018[19]	GP	755/3523	1.39 (1.27, 1.52)	per 1 SD increase in rank normalized data	NA	2.18	2.05 (1.69, 2.50)	+++
Okuno, 2009[35]	HD patients	91/ 490	1.05 (1.01, 1.08)	per 1 mg/L increase (0.14 SD)	NA	15.71	2.15 (1.27, 3.64)	++++
Liabeuf, 2012[29]	CKD stage 1-5	44/ 142	4.11 (2.00, 8.43)	B2M >8.34 versus ≤8.34 mg/L	NA	1.34	6.61 (2.53, 17.28)	++++
Astor, 2013[36]	KTR	452/ 2190	4.57 (2.79, 7.48)	highest versus lowest fifth	NA	0.78	3.27 (2.23, 4.80)	+++++[f]
Foster, 2016[30]	CKD stage 1-3	653/ 3613[b]	1.93 (1.71, 2.18)	1/B2M, per unit decrease in z score (1 SD in B2M)	NA	2.18	4.20 (3.22, 5.47)	+++++
Estimates with maximum adjustment [g]:		*NR/ 1324*	*1.59 (1.13, 2.25)*	*1/B2M, per unit decrease in z score (1 SD in B2M)*	*NA*	*2.18*	*2.75 (1.30, 5.83)*	*Plus mGFR*
Wu, 2017[31]	CKD stage 3-5	29/ 312	21.52 (2.06, 225.05)	highest tertile versus lowest tertile	NR	1.00	21.52 (2.06, 224.93)	+++++
Yamashita, 2018[37]	HD patients	66/ 307	1.06 (1.02, 1.10)	per 1 mg/L increase (0.16 SD)	NA	13.96	2.26 (1.33, 3.82)	+++++
Chang, 2019[38]	PD patients	258/ 725	1.03 (0.72, 1.49)	highest tertile versus middle tertile	NR	2.00	1.06 (0.51, 2.20)	++++
Estimates with maximum adjustment [g]:		*258/ 725*	*0.87 (0.58, 1.31)*	*highest tertile versus middle tertile*	*NR*	*2.00*	*0.76 (0.34, 1.71)*	*Plus eGFRcr, PD factors*
**Infectious Mortality Studies**								
Cheung, 2008[34]	HD patients	180/ 1813	1.21 (1.07, 1.37)	per 10 mg/L increase (0.84 SD)	NA	2.60	1.64 (1.19, 2.26)	+++++
Astor, 2013[36]	KTR	98/ 2190	5.32 (3.43, 8.28)[d]	highest versus lowest fifth	NR	0.78	3.68 (2.61, 5.19)	+++++[f]
Chang, 2019[38]	PD patients	86/ 725	1.98 (1.00, 3.93)	highest tertile versus middle tertile	NR	2.00	3.92 (1.00, 15.41)	++++
Estimates with maximum adjustment [g]:		*86/ 725*	*2.05 (0.96, 4.38)*	*highest tertile versus middle tertile*	*NR*	*2.00*	*4.20 (0.92, 19.17)*	*Plus eGFRcr, PD factors*
**Non-cardiovascular Mortality Studies**								
Okuno, 2009[35]	HD patients	55/ 490	1.06 (1.02, 1.10)	per 1 mg/L increase (0.14 SD)	NA	15.71	2.50 (1.38, 4.52)	++++

Note: HD/PD patients and those at CKD Stage 5 are normally ESRD patients.

B2M: Beta-2-microglobulin; CI: Confidence Interval; CKD: Chronic Kidney Disease; eGFRcr: estimated Glomerular Filtration Rate based on creatinine; ESRD: End Stage Renal Disease; HD: Hemodialysis; KTR: Kidney Transplant Recipients; mGFR: measured Glomerular Filtration Rate; NA: Not Applicable; NR: Not Reported; PD: Peritoneal Dialysis; RR: Relative Risk.

[a] To convert reported log RR to log RR in highest versus lowest third of the B2M distribution; [b] The sample size was different for all-cause mortality as it included those with prevalent CVD at baseline; [c] Pooled estimated from Quintile 5a, 5b and 5c; [d] Converted based on the HR of a doubling of B2M; [e] -no adjustment, + adjusted for age and/or sex, ++ age, sex, and non-lipid risk factors (e.g. race, medication use) , +++ adjusted for age, sex, diabetes, body mass index/ blood pressure/ smoking and/or lipid markers, ++++adjusted for preceding plus inflammatory markers; +++++adjusted for preceding plus urinary indices. [f] Adjustments do not include inflammatory markers. [g] RR adjusted for conventional cardiovascular risk factors was chosen in priority if more than one estimates were reported, followed by RR with maximum adjustment.

Supplementary Figure 1. Sensitivity analyses by using estimates with maximum adjustments for cardiovascular disease outcomes, infectious mortality as well as all-cause mortality.

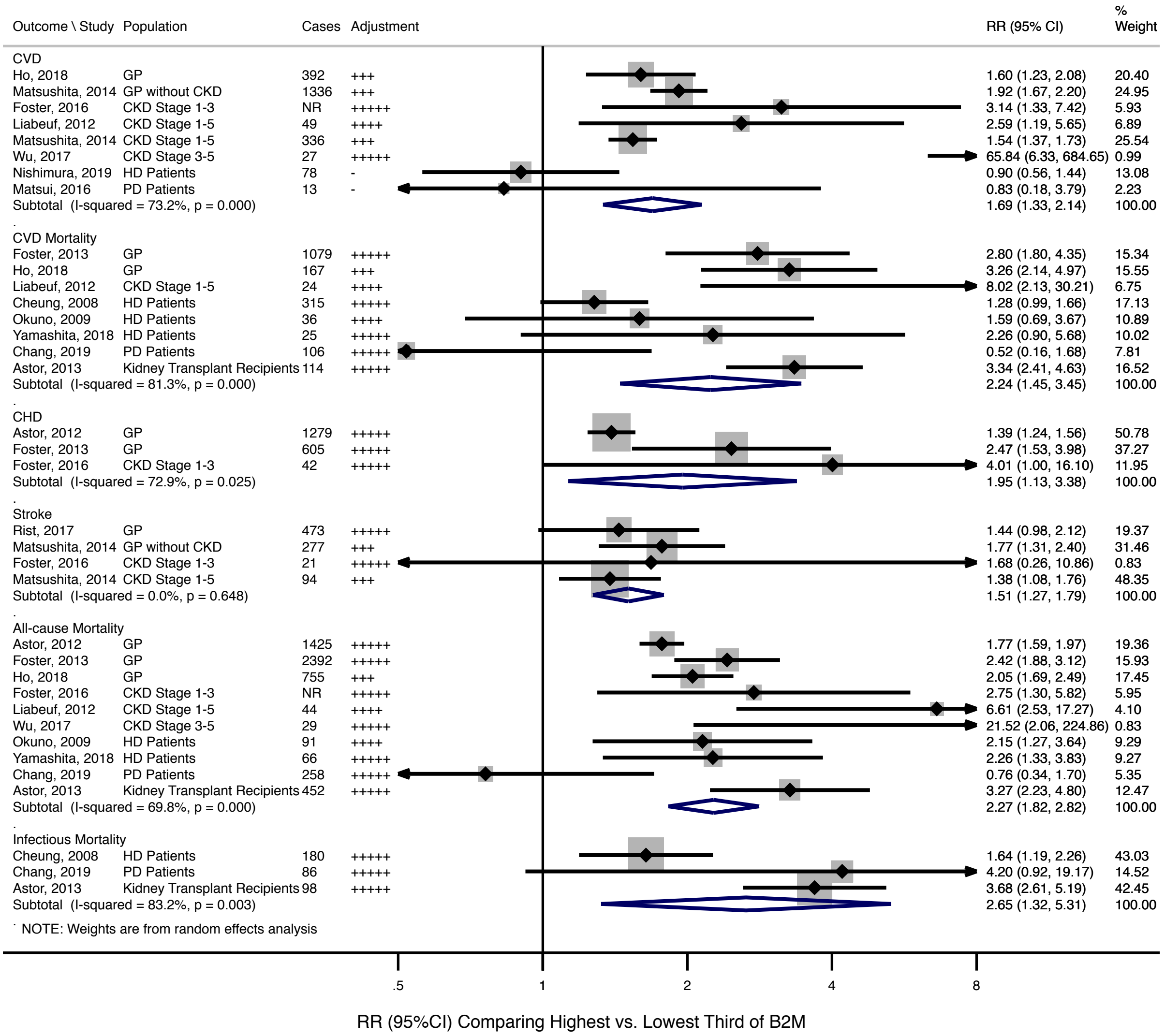

Note: HD/PD patients and those at CKD Stage 5 are normally ESRD patients.

B2M: Beta-2-microglobulin; CHD: Coronary Heart Disease; CI: Confidence Interval; CKD: Chronic Kidney Disease; CVD: Cardiovascular Disease; ESRD: End Stage Renal Disease; GP: General Populations; HD: Hemodialysis; PD: Peritoneal Dialysis; RR: Relative Risk.

Adjustment: -no adjustment, + adjusted for age and/or sex, ++ age, sex, and non-lipid risk factors (e.g. race, medication use) , +++ adjusted for age, sex, diabetes, body mass index/ blood pressure/ smoking and/or lipid markers, ++++adjusted for preceding plus inflammatory markers; +++++adjusted for preceding plus urinary indices.

Supplementary Figure 2. Sensitivity analyses by omitting one study at a time for (A) CVD, (B) CVD mortality and (C) all-cause mortality.

(A) CVD

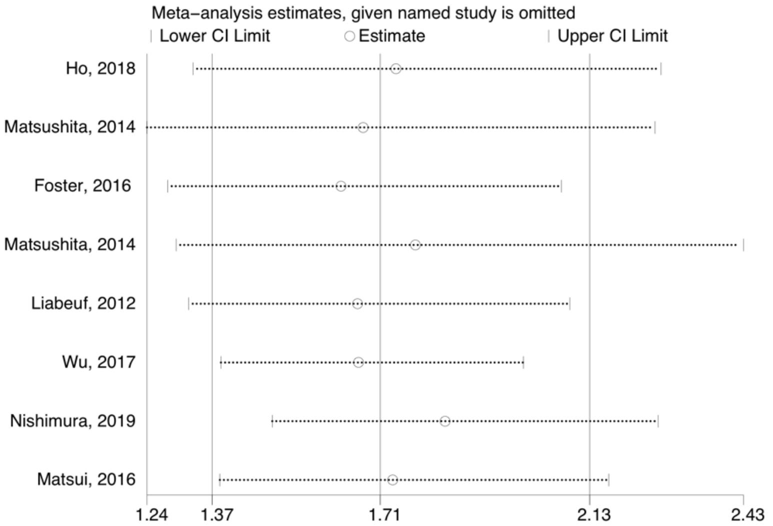

(B) CVD Mortality

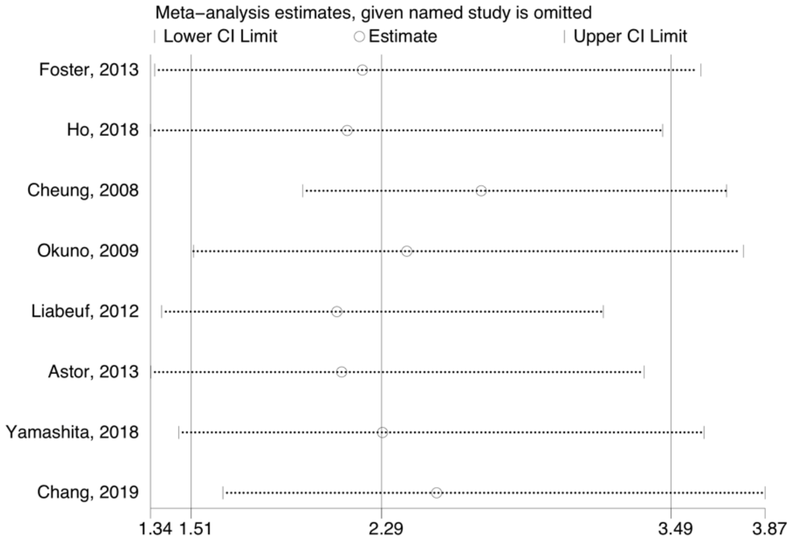

(C) All-cause Mortality

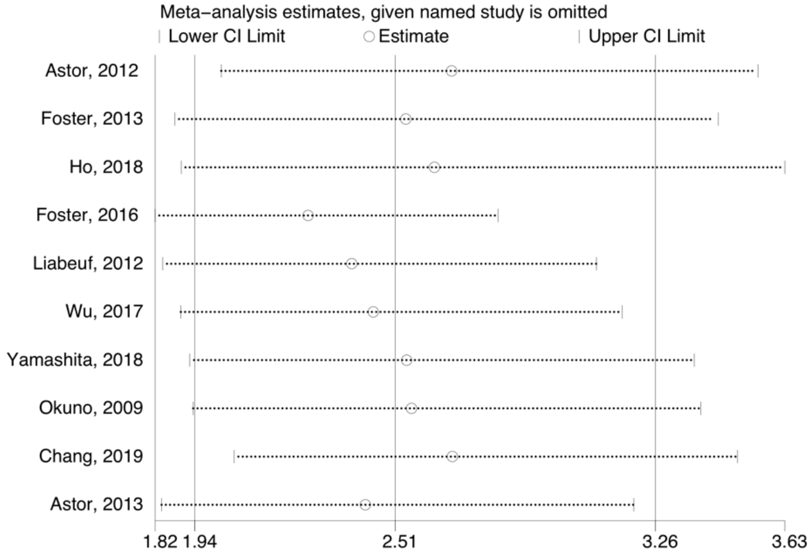

CVD: Cardiovascular Disease.

Supplementary Figure 3. Association of B2M with CHD, stroke, and all-cause mortality, restricted to participants without CKD.

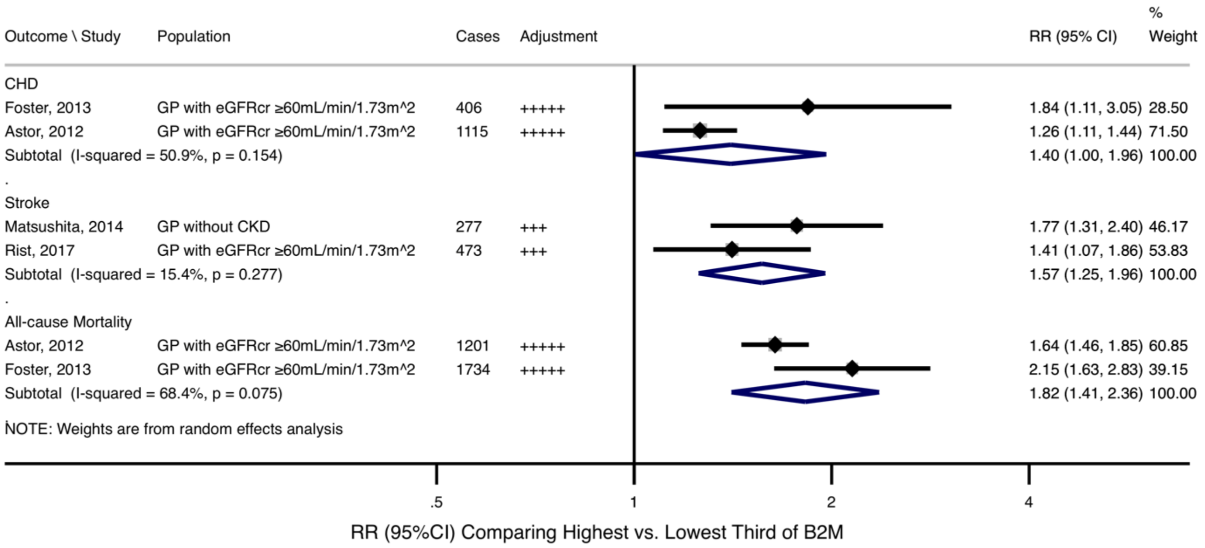

B2M: Beta-2-microglobulin; CHD: Coronary Heart Disease; CI: Confidence Interval; CKD: Chronic Kidney Disease; eGFRcr: estimated Glomerular Filtration Rate based on creatinine; GP: General Populations; RR: Relative Risk.

Adjustment: -no adjustment, + adjusted for age and/or sex, ++ age, sex, and non-lipid risk factors (e.g. race, medication use) , +++ adjusted for age, sex, diabetes, body mass index/ blood pressure/ smoking and/or lipid markers, ++++adjusted for preceding plus inflammatory markers; +++++adjusted for preceding plus urinary indices.

Supplementary Figure 4. Meta-regression on the effects of observation time and sample size for (A) CVD, (B) CVD mortality, and (C) all-cause mortality.

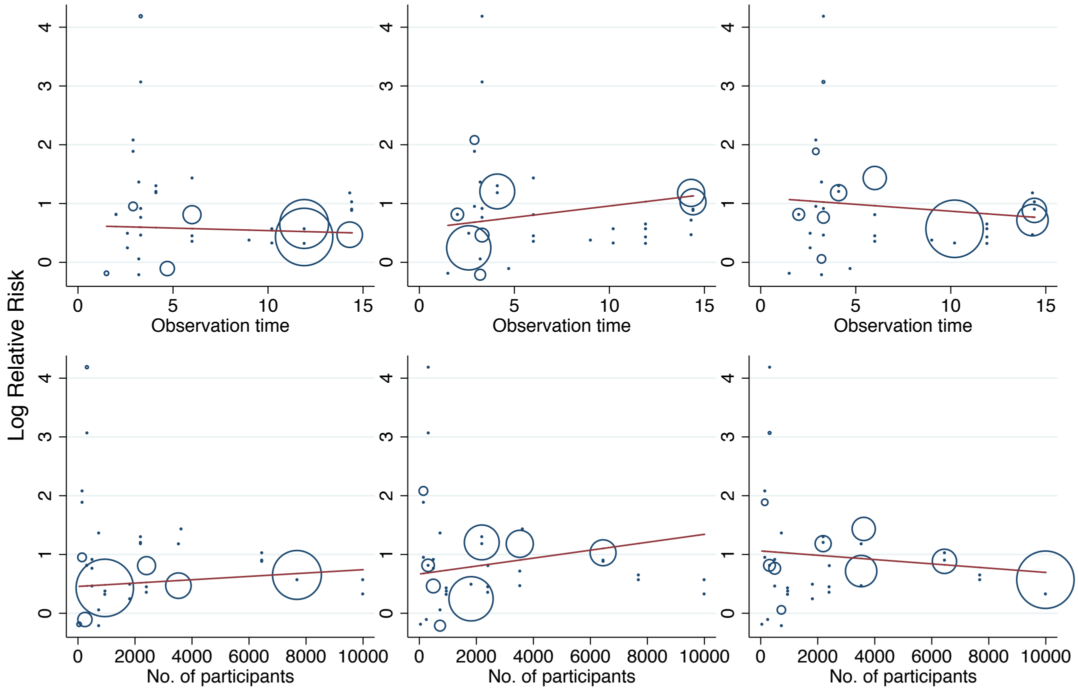

(A) CVD (B) CVD mortality (C) All-cause mortality

CVD: Cardiovascular Disease. All *p* >0.05.

Supplementary Figure 5. Funnel plot and filled funnel plot for studies of the association of B2M with CVD, CVD mortality and all-cause mortality.

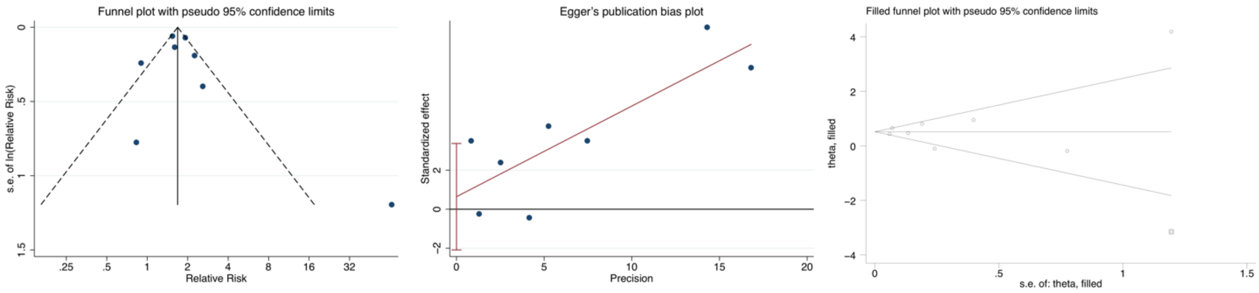

(A) CVD (Egger's test *p*= 0.587, Begg's test *p*=0.711)

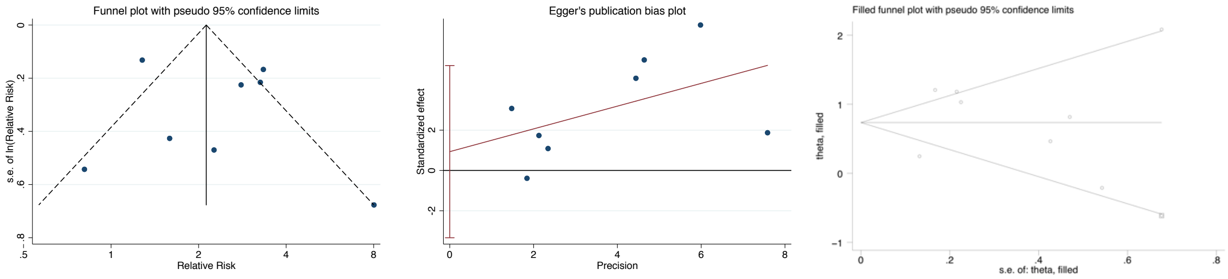

(B) CVD Mortality (Egger's test *p*= 0.612, Begg's test *p*=0.711)

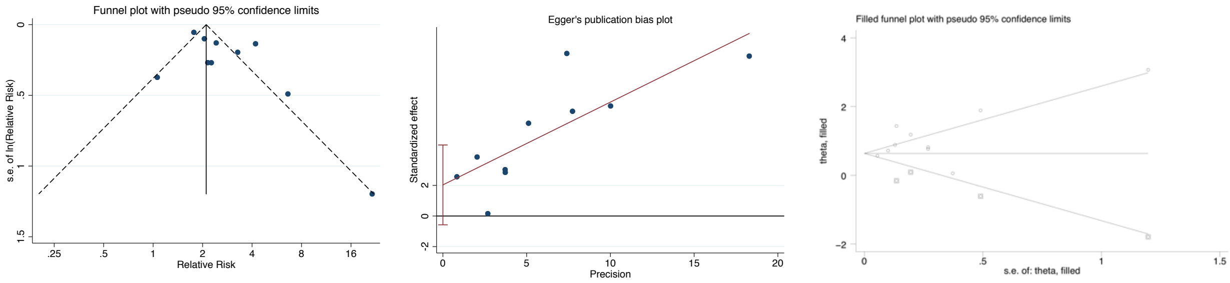

(C) All-cause Mortality (Egger's test *p*= 0.110, Begg's test *p*=0.371)

(Left) Funnel plot with 95% confidence intervals (CIs); (Middle) Egger's publication bias plot; (Right) Trimmed and filled funnel plot with pseudo-95% CIs. B2M: Beta-2-microglobulin; CI: Confidence Interval; CVD: Cardiovascular disease.
